# Systematic analysis of the basic/helix-loop-helix (bHLH) transcription factor family in pummelo (*Citrus grandis*) and identification of the key members involved in the response to iron deficiency

**DOI:** 10.1186/s12864-020-6644-7

**Published:** 2020-03-14

**Authors:** Xiao-Yong Zhang, Jie-Ya Qiu, Qiu-Ling Hui, Yuan-Yuan Xu, Yi-Zhong He, Liang-Zhi Peng, Xing-Zheng Fu

**Affiliations:** 1 Citrus Research Institute, Southwest University, Chongqing, 400712 China; 2 Citrus Research Institute, National Citrus Engineering Research Center, Chinese Academy of Agricultural Sciences, Chongqing, 400712 China

**Keywords:** Citrus, bHLH, Stress, Motif, Gene expression

## Abstract

**Background:**

Iron (Fe) deficiency is a common problem in citrus production. As the second largest superfamily of transcription factors (TFs), the basic/helix-loop-helix (bHLH) proteins have been shown to participate in the regulation of Fe homeostasis and a series of other biological and developmental processes in plants. However, this family of members in citrus and their functions in citrus Fe deficiency are still largely unknown.

**Results:**

In this study, we identified a total of 128 CgbHLHs from pummelo (*Citrus grandis*) genome that were classified into 18 subfamilies by phylogenetic comparison with *Arabidopsis thaliana* bHLH proteins. All of these *CgbHLHs* were randomly distributed on nine known (125 genes) and one unknown (3 genes) chromosomes, and 12 and 47 of them were identified to be tandem and segmental duplicated genes, respectively. Sequence analysis showed detailed characteristics of their intron-exon structures, bHLH domain and conserved motifs. Gene ontology (GO) analysis suggested that most of *CgbHLHs* were annotated to the nucleus, DNA-binding transcription factor activity, response to abiotic stimulus, reproduction, post-embryonic development, flower development and photosynthesis. In addition, 27 CgbHLH proteins were predicted to have direct or indirect protein-protein interactions. Based on GO annotation, RNA sequencing data in public database and qRT-PCR results, several of *CgbHLHs* were identified as the key candidates that respond to iron deficiency.

**Conclusions:**

In total, 128 CgbHLH proteins were identified from pummelo, and their detailed sequence and structure characteristics and putative functions were analyzed. This study provides comprehensive information for further functional elucidation of *CgbHLH* genes in citrus.

## Background

Citrus is the largest fruit crop in the world, which provides not only necessary nutrition for human but also substantial economic income for farmers. In 2015, the global citrus area was 13.5 million hectares and the yield reached 178.2 million tons (FAO statistics, http://faostat.fao.org/default.aspx). However, citrus production is also continuously influenced by many environmental factors, such as diseases, cold, drought, heat, and nutrient disorders, among which iron (Fe) deficiency is a common problem that can cause severe chlorosis of leaves, impaired tree vigor, and reduction of fruit yield and quality in citrus production [1]. In particular, in calcareous soils, citrus plants are highly sensitive to low Fe availability because bioavailable forms of ferrous Fe (II) are oxidized into insoluble ferric Fe (III) in a high-pH and oxygen-rich environment [2, 3]. To cope with this issue, plants have developed two major adaptive mechanisms for efficient Fe uptake from soils. All dicot and non-graminaceous monocot plants, such as *Arabidopsis thaliana* (*A. thaliana*), use a reduction-based strategy (strategy I), while graminaceous plants such as rice employ a chelation-based strategy (strategy II) [3–5]. In both strategy I and strategy II, one class of transcription factors (TFs), the basic/helix-loop-helix genes (*bHLHs*), is found to play the core regulatory role [3, 4, 6–9].

The first discovery of bHLH was during the study of murine muscle development [10]. Thereafter, the proteins containing this conserved domain have been widely identified in all three eukaryotic kingdoms and constitute one of the largest families of TFs [11–15]. In plants, the bHLH proteins are recognized as the second largest superfamily of TFs [12]. The bHLH domain consists of approximately 60 amino acids with two functionally distinct regions, the basic region and the HLH region [12, 14]. The basic region, containing 13 to 17 amino acids, is located at the N-terminus of the bHLH domain and functions in specific recognition and binding to the DNA motif of the target gene promoter [16]. In contrast, the HLH region is located at the C-terminal end and consists of approximately 40–50 amino acids with two amphipathic α helices that are linked by a loop of variable length. This region often functions in domain dimerization and allows the formation of homodimers or heterodimers to promote protein-protein interactions [12, 16, 17]. Outside of the conserved bHLH domain, the sequence of bHLH proteins is considerably divergent [18].

As an essential superfamily of TFs, bHLH proteins participate in regulating of a series of biological and developmental processes, such as flowering [19], root development [20], seed germination [21], anthocyanin or flavonoid metabolism [22–24], hormonal signaling regulation [25, 26], as well as biotic and abiotic stresses responses [14, 27–29]. Under Fe deficiency stress, bHLHs have been shown to play a predominant regulatory role. The FER-like iron-deficiency-induced transcription factor (FIT), encoding a bHLH29 orthologous to the tomato FER protein, is the first identified TF that regulates Fe homeostasis in *A. thaliana* [30–32]. Subsequently, bHLH38, bHLH39, bHLH100, and bHLH101, belonging to the Ib subgroup of the bHLH, are found to form heterodimers with FIT and positively regulate the expression of *IRT1* (iron-regulated transporter 1) and *FRO2* (ferric reduction oxidase 2) under Fe deficiency [9, 33]. POPEYE (PYE), another bHLH protein (bHLH47), acts as a negative regulator to participate in Fe homeostasis [34]. Recently, bHLH34, bHLH104, bHLH105, and bHLH115 have been shown to play essential roles in Fe homeostasis by positively regulating bHLH38, bHLH39, bHLH100, bHLH101 and PYE [8, 35, 36]. Moreover, four IVa subgroups of bHLH members, bHLH18, bHLH19, bHLH20, and bHLH25, were identified as novel interactors of FIT and mediate jasmonic acid-induced FIT protein degradation under Fe deficiency [6].

Although many *bHLH* genes in response to Fe deficiency have been well documented in model plants, it is still poorly understood in citrus. Pummelo (*Citrus grandis*) is a major cultivated species of citrus, which also belongs to the progenitor species that contributes to the generation of hybridized mandarins and sweet orange [37]. Both directly cultivated pummelos and the varieties grafted on pummelo rootstocks readily show Fe deficiency. Recently, high quality genome information of pummelo [38] was published, which promoted us to consider that we can use pummelo as material to systematically analyze the bHLH family genes and identify the essential members involved in the response to Fe deficiency in citrus. Therefore, in the present study, we identified 128 bHLH family members from pummelo at the whole genome level. We also carried out a detailed analysis and prediction of their sequence characteristics, phylogeny, gene duplication, chromosomal distribution, gene structure, protein motif, and protein-protein interaction. The bHLH members in response to Fe deficiency were identified by performing gene ontology (GO) annotation and time-course expression analysis under Fe deficiency. Our results provide valuable clues for functional elucidation of the *bHLH* genes in citrus, especially for revealing the regulatory mechanisms of *bHLHs* in citrus under Fe deficiency in the future.

## Results

### Identification and classification of CgbHLH members in pummelo

**Fig. 1:**
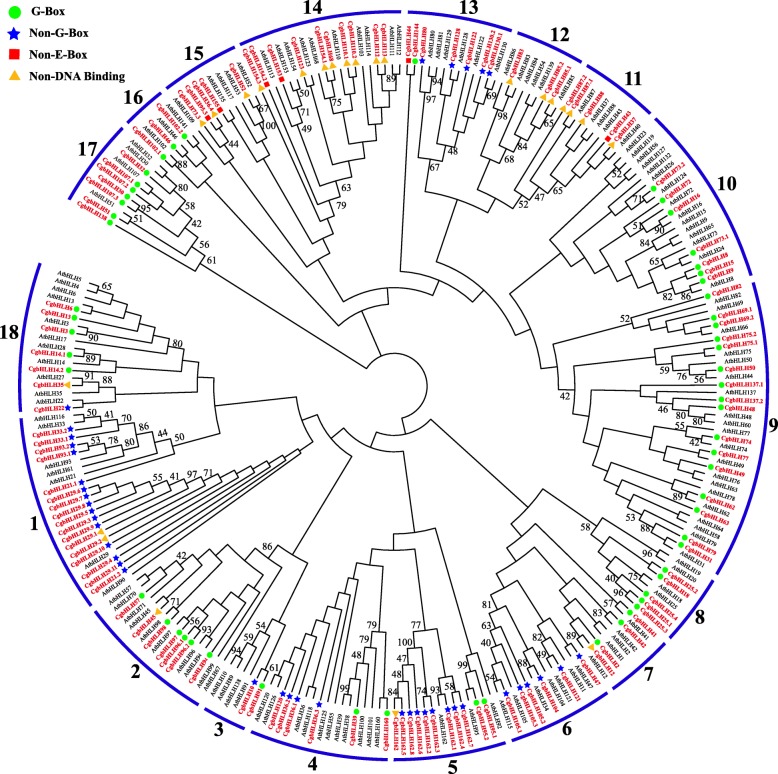
Phylogenetic tree and subfamily classifications of CgbHLH and AtbHLH proteins. The conserved protein sequences of a total of 128 CgbHLHs and 136 AtbHLHs were used to construct neighbor-joining phylogenetic tree. Arabic numerals 1 to 18 represent 18 of subfamilies. All CgbHLH proteins are highlighted with red color. The G-box, non-G-box, non-E-box and non-DNA binding of CgbHLHs are marked with different colored symbols

Based on the methods in “Materials and methods,” we finally identified 128 bHLH proteins from pummelo (Table 1 and Additional file 2: Table S1). These bHLHs showed 21.48% to 73.16% sequence identity with bHLHs of *A. thaliana* (AtbHLHs), and they were named CgbHLHs according to the highest sequence identity of AtbHLH (Table 1). If more than one CgbHLHs corresponded to the same AtbHLH, they were presented with an extra decimal point (e.g. CgbHLH29.1–CgbHLH29.11). The ORF length of the *CgbHLHs* ranged from 210 bp (*CgbHLH29.1*) to 4401 bp (*CgbHLH73.2*), encoding 69–1466 amino acids (Table 1 and Additional file 2: Table S1). Although these *CgbHLHs* showed large differences in length, 74.2% of them (95/128) were in the range of 700–2000 bp. The predicted MW and pI of CgbHLHs ranged from 7.56 to 163.79 kDa and 4.55 to 10.41, respectively (Additional file 2: Table S1). To explore the evolutionary relationship and the classification of CgbHLHs, a neighbor-joining phylogenetic tree was constructed with conserved sequences of 128 CgbHLHs and 136 AtbHLHs. Based on this phylogenetic analysis and the previous reported classification of the AtbHLHs, we classified 128 CgbHLHs into 18 subfamilies using Arabic numerals 1–18 (Fig. 1). Group 1 was the largest subfamily with 17 CgbHLH members, while the smallest, group 3, contained only 2 members.

**Table 1:** Summary of 128 identified CgbHLH members

No.	Gene Name	ORF Length	Gene ID	*A. thaliana* Homologs	Abbr. Name	Identity%	No.	Gene Name	ORF Length	Gene ID	*A. thaliana* Homologs	Abbr. Name	Identity%
1	CgbHLH2	1869	Cg5g002660	AT1G63650	EGL3	52.09	65	CgbHLH73.3	915	Cg1g019630	AT5G67110	ALC	26.42
2	CgbHLH3	1512	Cg5g034370	AT4G16430	JAM3	58.3	66	CgbHLH74	1284	Cg3g013130	AT1G10120	CIB4	49.3
3	CgbHLH6	2064	CgUng000770	AT1G32640	JAI1	61.74	67	CgbHLH75.1	717	Cg2g036150	AT1G25330	CES	52.2
4	CgbHLH8	2229	Cg5g013200	AT1G09530	PIF3	44.38	68	CgbHLH75.2	1095	Cg6g019500	AT1G25330	CES	39.9
5	CgbHLH9	1632	Cg7g012190	AT2G43010	PIF4	44.07	69	CgbHLH77	1455	Cg9g014180	AT3G23690	CIL2	47.21
6	CgbHLH10	1071	Cg9g023740	AT2G31220	–	46.5	70	CgbHLH79	840	Cg5g004140	AT5G62610	–	59.71
7	CgbHLH12	1974	Cg5g042050	AT4G00480	MYC1	49.49	71	CgbHLH80	765	Cg3g020720	AT1G35460	FBH1	63.68
8	CgbHLH13	1482	Cg5g040610	AT1G01260	JAM2	57.11	72	CgbHLH82	1563	Cg4g018170	AT5G58010	LRL3	43.38
9	CgbHLH14.1	1551	Cg5g040200	AT4G00870	–	47.04	73	CgbHLH83	888	Cg6g018550	AT1G66470	RHD6	48.46
10	CgbHLH14.2	1563	Cg5g000450	AT4G00870	–	37.07	74	CgbHLH85.1	1089	Cg8g003130	AT4G33880	RSL2	51.39
11	CgbHLH15	1764	Cg5g032930	AT2G20180	PIL5	46.61	75	CgbHLH85.2	1104	Cg3g012730	AT4G33880	RSL2	45.74
12	CgbHLH16	1386	Cg5g043840	AT4G00050	UNE10	59.43	76	CgbHLH87.1	1296	Cg1g009170	AT3G21330	–	52.65
13	CgbHLH18	783	Cg5g017710	AT2G22750	–	41.44	77	CgbHLH87.2	513	Cg6g009450	AT3G21330	–	47.87
14	CgbHLH21.1	1785	Cg8g001500	AT2G16910	AMS	48.72	78	CgbHLH88	822	Cg3g021250	AT5G67060	HEC1	50.93
15	CgbHLH21.2	1419	Cg7g019250	AT2G16910	AMS	36.78	79	CgbHLH91	1428	Cg5g045050	AT2G31210	–	45.32
16	CgbHLH22	585	Cg2g040910	AT4G21330	DYT1	43.92	80	CgbHLH93.1	1065	Cg1g007400	AT5G65640	NFL	52.13
17	CgbHLH25.1	570	Cg8g002160	AT4G37850	–	49.25	81	CgbHLH93.2	1155	Cg3g013530	AT5G65640	NFL	46.11
18	CgbHLH25.2	1017	Cg1g007240	AT4G37850	–	44.83	82	CgbHLH94	957	Cg1g008260	AT1G22490	–	46.89
19	CgbHLH25.3	426	Cg8g002170	AT4G37850	–	44.56	83	CgbHLH95.1	939	Cg8g004350	AT1G49770	RGE1	42.18
20	CgbHLH25.4	1107	Cg1g007260	AT4G37850	–	44.04	84	CgbHLH95.2	804	Cg3g021160	AT1G49770	RGE1	41.02
21	CgbHLH29.1	210	Cg8g019060	AT2G28160	FIT	64.41	85	CgbHLH95.3	729	Cg2g044520	AT1G49770	RGE1	24.66
22	CgbHLH29.2	222	Cg8g019160	AT2G28160	FIT	61.9	86	CgbHLH96.1	993	Cg3g014510	AT1G72210	–	56.62
23	CgbHLH29.3	489	Cg8g018990	AT2G28160	FIT	52	87	CgbHLH96.2	906	Cg2g030740	AT1G72210	–	51.72
24	CgbHLH29.4	969	Cg8g019240	AT2G28160	FIT	51.16	88	CgbHLH97	1287	Cg9g012540	AT3G24140	FMA	60.67
25	CgbHLH29.5	501	Cg8g019040	AT2G28160	FIT	50.49	89	CgbHLH98	966	Cg9g028240	AT5G53210	SPCH	61.29
26	CgbHLH29.6	633	Cg8g019200	AT2G28160	FIT	49.54	90	CgbHLH102.1	1029	Cg7g015780	AT1G69010	BIM2	60.19
27	CgbHLH29.7	237	Cg8g019020	AT2G28160	FIT	49.25	91	CgbHLH102.2	1026	Cg6g020770	AT1G69010	BIM2	44.37
28	CgbHLH29.8	660	Cg8g019140	AT2G28160	FIT	48.15	92	CgbHLH104	648	Cg9g007860	AT4G14410	–	64.62
29	CgbHLH29.9	651	Cg8g018940	AT2G28160	FIT	47.27	93	CgbHLH105.1	714	Cg2g023210	AT5G54680	ILR3	73.16
30	CgbHLH29.10	1416	Cg8g019230	AT2G28160	FIT	37.17	94	CgbHLH105.2	711	Cg2g017780	AT5G54680	ILR3	65.18
31	CgbHLH29.11	903	Cg8g018890	AT2G28160	FIT	33.15	95	CgbHLH105.3	711	Cg2g040740	AT5G54680	ILR3	62.11
32	CgbHLH30	798	Cg2g043260	AT1G68810	ABS5	41.67	96	CgbHLH107.1	717	Cg6g017840	AT3G56770	–	59.26
33	CgbHLH31	753	Cg7g020320	AT1G59640	ZCW32	51.95	97	CgbHLH107.2	786	Cg3g014080	AT3G56770	–	37.04
34	CgbHLH32	789	Cg7g014890	AT3G25710	TMO5	58.65	98	CgbHLH107.3	846	Cg2g008600	AT3G56770	–	35.87
35	CgbHLH33.1	1659	Cg7g011080	AT1G12860	ICE2	57.28	99	CgbHLH110.1	1299	Cg3g023160	AT1G27660	–	51.35
36	CgbHLH33.2	1443	Cg2g009820	AT1G12860	ICE2	55.8	100	CgbHLH110.2	1104	CgUng019530	AT1G27660	–	48.1
37	CgbHLH35	747	Cg4g021000	AT5G57150	–	57.2	101	CgbHLH112	1479	Cg2g040950	AT1G61660	–	44.04
38	CgbHLH36.1	741	Cg9g025850	AT5G51780	–	53.85	102	CgbHLH113	819	Cg8g003580	AT3G19500	–	45.71
39	CgbHLH36.2	744	Cg5g009400	AT5G51780	–	42.77	103	CgbHLH120	537	Cg9g025860	AT5G51790	–	47.46
40	CgbHLH36.3	726	Cg9g025870	AT5G51780	–	38.69	104	CgbHLH121	1029	CgUng005820	AT3G19860	–	61.39
41	CgbHLH36.4	1122	Cg3g014280	AT5G51780	–	25.81	105	CgbHLH122	1296	Cg2g002540	AT1G51140	FBH3	45.14
42	CgbHLH37	807	Cg1g002590	AT3G50330	HEC2	53.88	106	CgbHLH123	1377	Cg2g022720	AT3G20640	–	49.59
43	CgbHLH39	762	Cg9g016290	AT3G56980	ORG3	47.5	107	CgbHLH128	1116	Cg5g028960	AT1G05805	AKS2	55.06
44	CgbHLH41	1599	Cg4g008800	AT5G56960	–	29.22	108	CgbHLH130.1	2334	Cg9g022410	AT2G42280	FBH4	57.14
45	CgbHLH42	2073	Cg5g035630	AT4G09820	TT8	56.51	109	CgbHLH130.2	1287	Cg7g023040	AT2G42280	FBH4	45.99
46	CgbHLH43	462	Cg1g025450	AT5G09750	HEC3	66.92	110	CgbHLH137.1	1116	Cg7g010980	AT5G50915	–	45.96
47	CgbHLH44	1200	Cg1g004400	AT1G18400	BEE1	21.48	111	CgbHLH137.2	837	Cg7g022310	AT5G50915	–	43.89
48	CgbHLH45	555	Cg9g004620	AT3G06120	MUTE	65.03	112	CgbHLH138	1542	Cg1g007950	AT2G31215	–	28
49	CgbHLH46	1761	Cg5g012000	AT5G08130	BIM1	53.08	113	CgbHLH144	609	Cg1g004390	AT1G29950	SACL3	23.28
50	CgbHLH47	729	Cg5g003170	AT3G47640	PYE	51.79	114	CgbHLH153	435	Cg5g026370	AT1G05710	–	66.42
51	CgbHLH48	1227	Cg9g022350	AT2G42300	–	59.32	115	CgbHLH154.1	933	Cg1g012580	AT2G31730	–	47
52	CgbHLH49	1680	Cg7g015600	AT1G68920	CIB1	54.17	116	CgbHLH154.2	735	Cg7g004740	AT2G31730	–	36.36
53	CgbHLH50	798	Cg5g022610	AT1G73830	BEE3	55.27	117	CgbHLH154.3	1107	Cg5g035790	AT2G31730	–	24.68
54	CgbHLH51	843	Cg7g002190	AT2G40200	–	42.34	118	CgbHLH155	2250	Cg5g034200	AT2G31280	LHL2	47.44
55	CgbHLH52	939	Cg2g038660	AT1G30670	–	42.51	119	CgbHLH160	999	Cg3g004340	AT1G71200	CITF1	40.58
56	CgbHLH57	1299	Cg5g039930	AT4G01460	–	51.96	120	CgbHLH162.1	570	Cg2g042210	AT4G20970	–	42.93
57	CgbHLH62	1665	Cg6g008950	AT3G07340	CIB3	53.27	121	CgbHLH162.2	345	Cg2g005390	AT4G20970	–	38.16
58	CgbHLH63	1215	Cg8g007040	AT4G34530	CIB1	46.28	122	CgbHLH162.3	279	Cg2g005350	AT4G20970	–	37.84
59	CgbHLH68	1023	Cg1g014350	AT4G29100	–	51.43	123	CgbHLH162.4	549	Cg5g004810	AT4G20970	–	35.23
60	CgbHLH69.1	633	Cg3g010300	AT4G30980	LRL2	55.43	124	CgbHLH162.5	498	Cg2g005320	AT4G20970	–	33.54
61	CgbHLH69.2	1044	Cg4g024560	AT4G30980	LRL2	52.04	125	CgbHLH162.6	462	Cg2g005400	AT4G20970	–	33.56
62	CgbHLH72	1155	Cg5g010830	AT5G61270	PIF7	47.52	126	CgbHLH162.7	666	Cg2g015830	AT4G20970	–	31.28
63	CgbHLH73.1	1233	Cg1g005520	AT5G67110	ALC	49.15	127	CgbHLH162.8	498	Cg2g005380	AT4G20970	–	31.06
64	CgbHLH73.2	4401	Cg5g039380	AT5G67110	ALC	29.05	128	CgbHLH162.9	420	Cg2g005340	AT4G20970	–	33.33

Abbreviation (Abbr.) names are the known gene names in *A. thaliana*, and their full names are shown in abbreviations list

### Multiple sequence alignment and analysis of the gene structure, conserved motif and domain of CgbHLHs

**Fig. 2:**
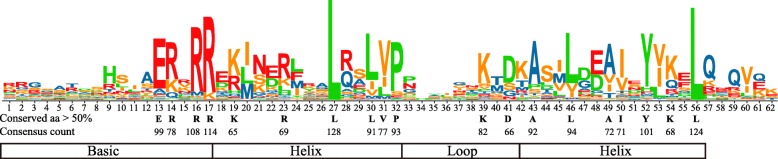
Conserved amino acids in CgbHLH domains. The height of each amino acid represents its conservation at that position. The amino acids conserved with more than 50% consensus ratio among the 128 CgbHLH domains are indicated in black letters

As shown in Additional file 1: Figure S1, multiple sequence alignment of 128 CgbHLHs showed that most of them were highly conserved in their bHLH domains, except that CgbLH37, CgbLH87.1, CgbLH88, CgbLH29.1, CgbLH45 and CgbLH162.9 were absent in the basic region, and CgbLH96.2 and CgbLH25.1 were short of the second helix. There were 19 conserved amino acid residues with a consensus ratio higher than 50%, including the basic region of Glu-13, Arg-14, Arg-16 and Arg-17, the first helix region of Lys-19, Arg-23, Leu-27, Leu-30, Val-31 and Pro-32, the loop region of Lys-39 and Asp-41, and the second helix region of Ala-43, Leu-46, Ala-49, Ile-50, Tyr-52, Lys-54 and Leu-56 (Fig. 2 and Additional file 1: Fig. S1). Among these, Arg-16, Arg-17, Leu-27 and Leu-56 showed extreme conservation, with a consensus ratio higher than 80%. The basic region of CgbHLHs consisted of a maximum of 17 amino acid residues, which determined the DNA binding ability of CgbHLH proteins. Based on the rule developed by Toledo-Ortiz et al. (2003) [6], we identified 104 DNA binding CgbHLHs (more than 5 basic residues existing in their basic region) and 24 non-DNA binding CgbHLHs (less than 6 basic residues existing in their basic region). The DNA binding CgbHLHs were further subdivided into 98 E-box binders (containing Glu-13 and Arg-16) and 6 non-E-box binders (lacking Glu-13 or Arg-16) based on the presence or absence of Glu-13 and Arg-16. Most of non-DNA binding and non-E-box CgbHLHs were distributed in subfamilies 11, 12, 14 and 15 (Fig. 1). Among 98 E-box CgbHLHs, 58 proteins (containing His/Lys-9, Glu-13 and Arg-17) were predicted to bind the G-box motif (CACGTG), while 40 proteins were predicted to recognize other types of E-boxes (CANNTG) and were defined as non-G-box binders (Fig. 1).

**Fig. 3:**
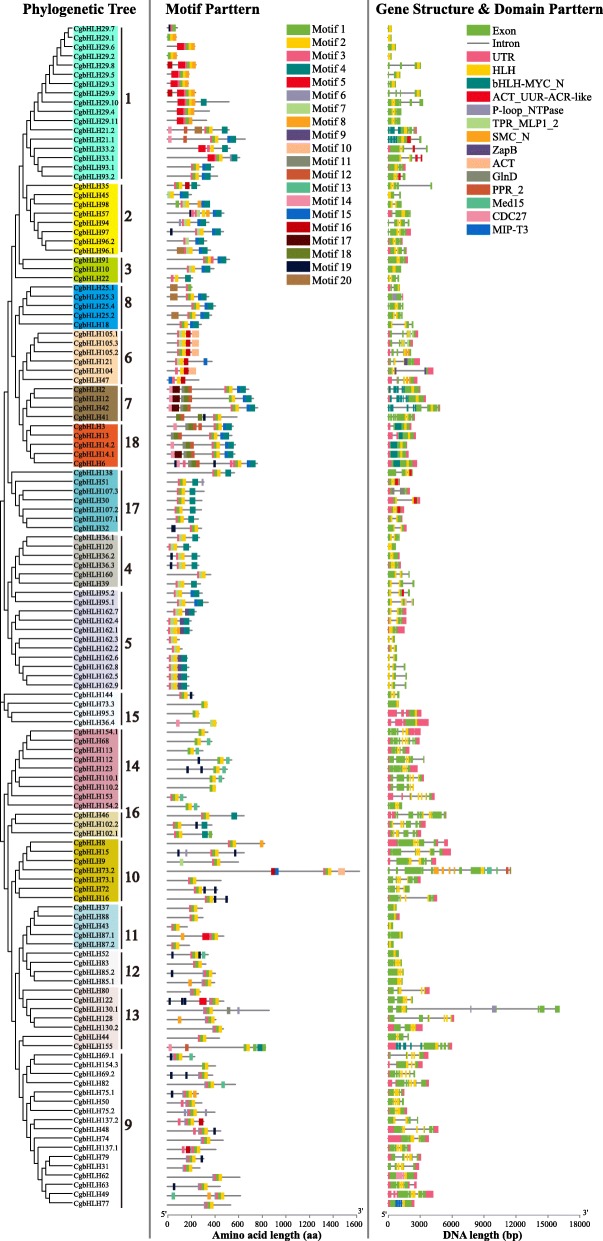
Gene structures and protein motifs of CgbHLHs. The intron-exon structures, predicted motifs 1 to 20, and conserved domains of all CgbHLHs are shown according to their phylogenic classifications

Gene structure analysis showed that 127 CgbHLHs contained 1 to 14 exons, and one CgbHLH (*CgbHLH73.2*) contained 21 exons (Additional file 2: Table S1). A total of 6 members (*CgbHLH37/43/87.2/88* in subfamily 11 and *CgbHLH6/14.1* in subfamily 18) were intronless, and 51 members had no untranslated region (UTR). Conserved motif prediction showed the constitutions of motif 1 to motif 20 in each CgbHLH protein (other possible motifs are not shown). Details of the 20 motifs are shown in Additional file 2: Table S1 and Fig. 3. Motifs 1, 2 and 3, located in bHLH domains, were found in almost all CgbHLHs, while the other motifs existed only in certain members. The members that phylogenetically clustered together or in the same subfamily often showed a similar gene structure and motif pattern. For example, the closely clustered pair CgbHLH93.1 and CgbHLH93.2 had identical motifs 1, 2, 3, 4 and 15. Most of the members of subfamily 1 contained motifs 1, 2, 3 and 4, and those of subfamily 2 contained motifs 4, 7 and 15. In addition, we found that motifs 9 and 10 were identified only in subfamily 5 and subfamily 6, respectively. Five types of motifs (motif 1, 3, 6, 18 and 19) were repeated twice or thrice in certain members, such as twice for motif 3 in CgbHLH48/50/57/137.1/137.2, twice for motif 6 in CgbHLH75.2, and thrice for motif 1 in CgbHLH155. As shown in Fig. 3, conserved domain analysis showed that all 128 CgbHLHs had an HLH domain, but there were also 36 *CgbHLH* genes with other domains, which might be fusion genes.

### Chromosomal distribution and gene duplication of CgbHLHs

**Fig. 4:**
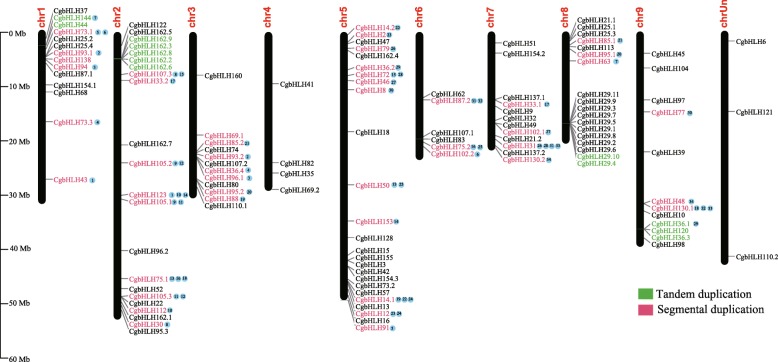
Chromosomal localizations and gene duplications of *CgbHLH* genes. The green and red colors represent tandem duplicated and segmental duplicated genes, respectively. The *CgbHLH* genes followed by the same number (highlighted with blue circles) are collinear genes

According to the genome annotation information, we found that 125 *CgbHLH* genes were distributed on nine chromosomes, while the chromosomal location of three *CgbHLHs* (*CgbHLH6/110.2/121*) could not be determined (Fig. 4). Chromosome 5 (chr5) contained the most *CgbHLHs* (25 genes), followed by chr2 (22 genes), chr8 (18 genes) and chr1 (14 genes). Although chr4 was longer than chr7 and chr8, it contained the fewest *CgbHLHs* (only 4 genes). The gene duplication and collinear correlations analysis showed that a total of 12 *CgbHLHs* were identified to be tandem duplicated genes, distributed on chr1, chr2, chr8 and chr9 (Fig. 4). In addition, 47 *CgbHLHs* were predicted to be segmental duplicated genes, accounting for about 37% of all *CgbHLH* genes, and the identified collinear genes showed great overlapping with these segmental duplicated genes (Fig. 4). These results suggested that the tandem and segmental duplication events that occurred during citrus evolution might have played an essential role in *CgbHLH* family expansion.

### GO annotation and protein interactions of CgbHLHs

**Fig. 5:**
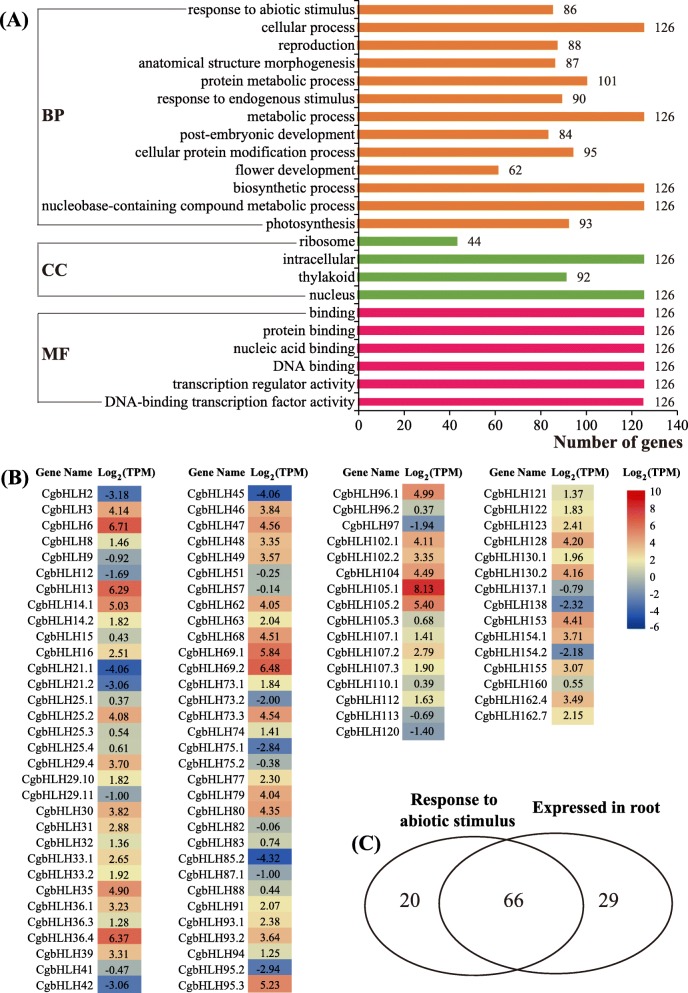
Functional annotation and tissue expression of *CgbHLH* genes. **a** All annotated GO terms including biological process (BP), cellular component (CC) and molecular function (MF) of 126 *CgbHLHs* (two other *CgbHLHs* were not annotated a GO term). **b** Expression heatmap of *CgbHLHs*. The TPM values were generated from the RNAseq data of pummelo roots in a public database. **c** Venn diagram shows the number of the genes that respond to abiotic stimulus and expresssed in roots

To determine potential functions of each *CgbHLH* gene, GO annotation was performed for all *CgbHLHs*. As shown in Fig. 5a, except for two genes that were not annotated a GO term, the other 126 *CgbHLHs* were annotated in three functional categories: biological process (BP), cellular component (CC) and molecular function (MF). In the BP category, these *CgbHLHs* were further annotated to respond to abiotic stimulus (86 genes), reproduction (88 genes), post-embryonic development (84 genes), flower development (62 genes) and photosynthesis (93 genes). Under CC and MF categories, we found that all 126 *CgbHLHs* were annotated to the nucleus, protein or DNA binding, and transcription factor activity, which agreed well with TF property of these *CgbHLHs*.

**Fig. 6:**
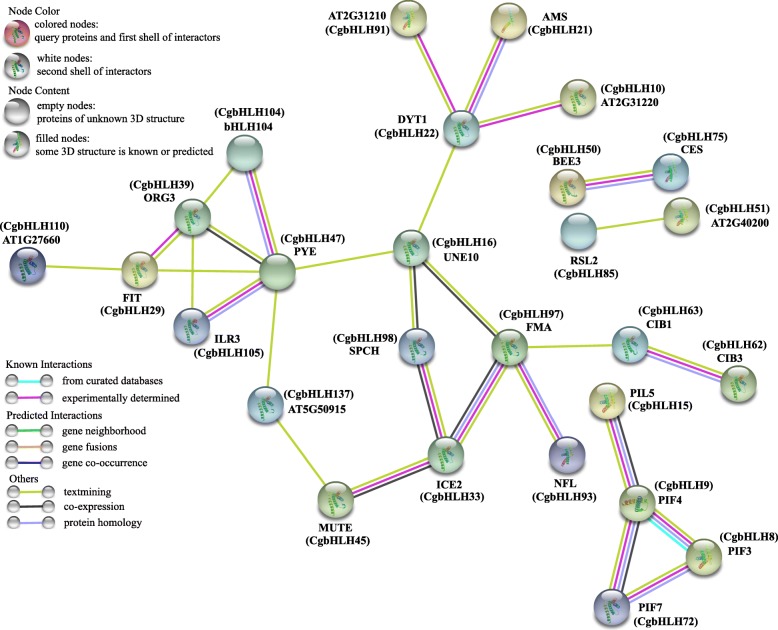
Predicted protein-protein interactions of CgbHLHs according to their orthologs in *A. thaliana*. In the network, only the pairs with higher than 40% sequence identity between CgbHLHs and AtbHLHs and with an interaction score > 0.7 are shown. Line and node colors indicate the different kinds and degrees of interactions, respectively. The filled or empty nodes represent known or unknown 3D structures, respectively. The abbreviated names are the genes that have been reported in *A. thaliana*

Proteins in the bHLH family often interact with each other by forming homodimers or heterodimers, which are essential for binding and regulating downstream target gene expression. Thus, prediction of protein interactions of CgbHLHs was performed by using orthologous bHLHs of *A. thaliana*. The result showed that a total of 27 CgbHLH proteins were predicted to have protein interaction relationships (Fig. 6). For example, CgbHLH29 (FIT ortholog), CgbHLH39, CgbHLH47 (PYE ortholog), CgbHLH104, CgbHLH105 (ILR3 ortholog) and CgbHLH110 were predicted to have direct or indirect interactions with each other. CgbHLH33 (ICE2 ortholog) was predicted to interact with CgbHLH45 (MUTE ortholog), CgbHLH97 (FMA ortholog) and CgbHLH98 (SPCH) directly. Moreover, interactions among several phytochrome interacting factor (PIF) orthologous proteins (CgbHLH8, CgbHLH9, CgbHLH15 and CgbHLH72) were predicted. Overall, the result provides an important reference for identifying true interactions of CgbHLHs with biochemical experiments.

### Candidate CgbHLH members in response to Fe deficiency

As described in the “Introduction,” *bHLH* genes play an essential regulatory role in plant Fe homeostasis. To identify candidate CgbHLH members in response to Fe deficiency in pummelo, their expression levels in the root were evaluated by analyzing the previous RNAseq data and performing qRT-PCR confirmation. Based on analysis of previous RNAseq data published by Guo et al. (2017) [39], a total of 95 *CgbHLH* genes were determined to be transcribed in the root of normal cultured pummelo (Fig. 5b). Their transcription levels (expressed as the TPM value) ranged from 0.05 (*CgbHLH85.2*) to 281.08 (*CgbHLH105.1*). These root-expressed genes are preliminarily considered to respond to Fe deficiency, as the root is directly responsible for ion uptake. To further narrow the range of candidates, we searched for root-expressed genes among the GO annotated abiotic stimulus responsive genes; as a result, 66 *CgbHLHs* were found to overlap (Fig. 5c). Due to lower sensitivity of qRT-PCR than RNAseq in determining gene expression, we finally selected 39 *CgbHLHs* with TPM values higher than 3.00 from these 66 genes for qRT-PCR confirmation. In addition, three genes (*CgbHLH104, CgbHLH105.1 and CgbHLH105.2*) that are orthologous to known Fe-related bHLHs but that were not included in the 39 *CgbHLHs* were also determined by qRT-PCR in this study.

**Fig. 7:**
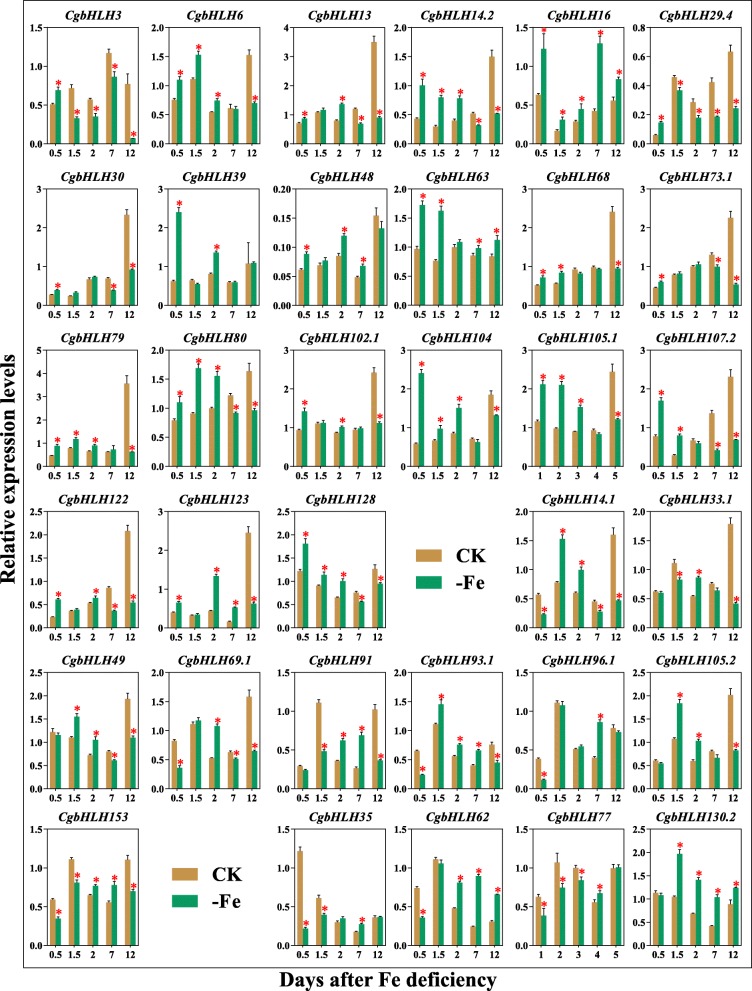
qRT-PCR analysis of 34 *CgbHLH* genes in pummelo roots. Relative expression levels of 34 *CgbHLHs* were determined in 0.5 d, 1.5 d, 2 d, 7 d and 12 d of Fe-deficient (−Fe) roots and normal (CK) roots sampled at corresponding time points. Data are means ± SE of three biological replicates. Asterisks on the error bars indicate significant differences (*t*-test, *P* < 0.05) between -Fe and CK

Except for eight genes were still undetectable, the other 34 genes were determined to be up- or down-regulated in roots of pummelo after 0.5 d, 1.5 d, 2 d, 7 d, and 12 d of Fe-deficient (−Fe) treatments when comparing with those at corresponding time points of CK (Fig. 7). In general, the tested genes showed three types of expression patterns. The first type, such as *CgbHLH3*, *CgbHLH6*, *CgbHLH13*, *CgbHLH14.2*, *CgbHLH16*, *CgbHLH29.4*, *CgbHLH30*, *CgbHLH39*, *CgbHLH48*, *CgbHLH63*, *CgbHLH68*, *CgbHLH73.1*, *CgbHLH79*, *CgbHLH80*, *CgbHLH102.1*, *CgbHLH104*, *CgbHLH105.1*, *CgbHLH107.2*, *CgbHLH122*, *CgbHLH123* and *CgbHLH128*, was up-regulated from an early period (0.5 d), which indicated an early response of these genes to Fe deficiency. The second type, such as *CgbHLH14.1*, *CgbHLH33.1*, *CgbHLH49*, *CgbHLH69.1*, *CgbHLH91*, *CgbHLH93.1*, *CgbHLH96.1*, *CgbHLH105.2* and *CgbHLH153*, was up-regulated at medium periods but was down-regulated during early and late periods. The third type was only up-regulated during late periods, such as *CgbHLH35*, *CgbHLH62*, *CgbHLH77* and *CgbHLH130.2*. Statistical analysis showed that most of the tested genes were significantly differentially expressed at multiple time points. In particular, *CgbHLH6*, *CgbHLH14.2*, *CgbHLH16*, *CgbHLH48*, *CgbHLH63*, *CgbHLH79*, *CgbHLH80*, *CgbHLH104*, *CgbHLH105.1*, *CgbHLH123*, *CgbHLH128*, *CgbHLH93.1*, *CgbHLH62* and *CgbHLH130.2* were significantly up-regulated while *CgbHLH3*, *CgbHLH29.4*, *CgbHLH14.1*, *CgbHLH69.1*, *CgbHLH153* and *CgbHLH77* were significantly down-regulated at least three time points. Moreover, *CgbHLH16* and *CgbHLH63* showed continuous up-regulation at all tested times. We speculate that these significantly differentially expressed *CgbHLH* genes are possibly the key candidates that respond to Fe deficiency in pummelo.

## Discussion

The genomes of several citrus varieties have been released, including the sweet orange (*Citrus sinensis*) genome published by Xu et al. (2011) [40], the clementine mandarin genome published by Wu et al. (2014) [41], and the pummelo, papeda (*Citrus ichangensis*) and citron (*Citrus medica*) genomes published by Wang et al. (2017) [38]. After the completion of these genomes, systematic identification and analysis of important gene families have been widely reported for citrus [1, 42–44]. These studies have provided us a comprehensive understanding of potential gene functions and have promoted related research progress. However, no such study has been done on the citrus bHLH family, except that Geng and Liu (2018) [45] identified 56 putative *bHLH* genes from sweet orange. In this study, we used a genome-wide approach to identify 128 CgbHLHs from pummelo. This number possibly indicates the real quantity of *bHLH* genes in most citrus varieties because the ratio of CgbHLHs in the pummelo genome (0.42%) is very similar to that of tomato (0.46%) [46], rice (0.44%) [13], poplar (0.40%) [11], apple (0.42%) [27], and grape (0.40%) [47]. With respect to the bHLHs of these plants, a high proportion of tandem duplications and segmental duplications has been determined, which indicates that bHLH expansion was possibly derived from gene duplication during evolution [48]. In citrus, an ancient whole-genome duplication event (WGD) occurred [40], and it could have led to this chromosome segmental duplication.

In the CgbHLH family, 19 amino acid residues are highly conserved, with a consensus ratio higher than 50%. These residues are also highly conserved in bHLHs of *A. thaliana* [15], maize [49] and grape [47], indicating conservation of bHLH families among different plants. Evidence has shown that at least five basic residues in the basic region of the bHLH domain determine DNA binding activity of bHLHs; Glu-13 is critical in specific recognition of the E-box DNA binding motif, while Arg-16 functions in fixing and stabilizing the position of Glu-13; moreover, His/Lys-9 and Arg-17 confer specificity to G-box (CACGTG) recognition [15]. Based on these findings, 128 CgbHLHs can be classified into four types: G-Box, non-G-Box, non-E-Box and non-DNA binding (Fig. 1). This classification indicates the basic functional property of each CgbHLH. We also noticed that Leu-27 was conserved in all CgbHLH proteins, and a similar result was found in AtbHLHs [15]. A previous study showed that the Leu residue at position 27 is necessary for dimer formation [50]. Thus, Leu-27 of CgbHLHs suggests their dimerization capacity.

To date, only very limited citrus bHLH genes have been functionally characterized, including *PtrbHLH*, an ortholog of *AtbHLH33* (ICE2), that was isolated from trifoliate orange (*Poncirus trifoliata*) and proved to confer cold tolerance by regulating both POD- and CAT-mediated reactive oxygen species (ROS) scavenging [29, 45]. *CsbHLH18* of sweet orange was also proved to modulate cold tolerance and ROS homeostasis [51]. Moreover, a *CubHLH1* of Satsuma mandarin (*Citrus unshiu* Marc.) was overexpressed in transgenic tomato fruit, resulting in modulation of carotenoid metabolism [52]. Generally, the potential functions of the unknown genes could be predicted through their orthologous genes. Based on phylogenetic and orthologous analyses, the CgbHLH family was classified into 18 subfamilies (Fig. 1). The closely clustered CgbHLHs and AtbHLHs in the same subfamily/group may have similar functions. In group 1, *AtbHLH29* (*FIT*), *AtbHLH33* (*ICE2*), *AtbHLH116* (*ICE1*), *AtbHLH93* (*NFL*)*, AtbHLH61,* and *AtbHLH21* (*AMS*) have been shown to be involved in Fe deficiency, cold tolerance and flower development [32, 53–55], suggesting that there are similar functions for the CgbHLHs of this group. In the other groups, many *AtbHLH* genes such as *AtbHLH70* (*MYC70*) and *AtbHLH45* (*MUTE*) in group 2*, AtbHLH10*, *AtbHLH89* and *AtbHLH91* in group 3, *AtbHLH38*, *AtbHLH39*, *AtbHLH100* and *AtbHLH101* in group 4, etc. have also been functionally characterized [9, 33, 56–58]. Based on these previous studies, the potential functions of most *CgbHLH* genes could be predicted. Furthermore, GO annotation and protein-protein interaction analysis of the *CgbHLHs* could help us to understand their possible functions.

Fe deficiency-regulated bHLHs are the main concerns in this study. qRT-PCR showed that most of the tested *CgbHLH* genes were significantly differentially expressed at two or more time points under Fe deficiency. Among them, *CgbHLH29.4, CgbHLH39*, *CgbHLH104, CgbHLH105.1* and *CgbHLH105.2* are orthologs of known Fe deficiency-responsive *AtbHLHs* [32, 33, 36], and their expression patterns further supported the putative function on Fe deficiency. *CgbHLH6*, *CgbHLH13*, *CgbHLH14.1* and *CgbHLH14.2* are orthologs of *AtHLH6* (*MYC2*), *AtHLH13* (*JAM2*) and *AtHLH14*, respectively, and these three *AtHLHs* of *A. thaliana* have been shown to participate in jasmonic acid (JA) signaling [6, 59]. Because JA is a negative regulator of Fe uptake, it suggests that these four *CgbHLH* genes may also be involved in the response to Fe deficiency. Interestingly, several of the significantly expressed *CgbHLHs* such as *CgbHLH105.1*, *CgbHLH104*, *CgbHLH13* and *CgbHLH130.2* also showed a high expression level in roots based on RNAseq data, which further indicates their possible functions in Fe deficiency. Moreover, we found that *CgbHLH104* and *CgbHLH105.1* belong to the same subfamily (Group 6) and have a similar gene structure, pI and exon number. Their orthologous *bHLHs* of *A. thaliana*, *AtbHLH104* and *AtbHLH105*, as well as all the other members in group 6 (*AtbHLH47* and *AtbHLH121*) have been functionally identified in Fe deficiency [7, 8, 34–36]. Herein, *CgbHLH104* and *CgbHLH105.1* also showed significant up-regulation under Fe deficiency. Thus, we speculate that these two *CgbHLHs* most likely have similar functions with *AtbHLH104* and *AtbHLH105* under Fe deficiency. Apart from these known Fe deficiency-responsive *bHLHs*, there were also significantly differentially expressed *CgbHLHs*, but direct evidence could not be found regarding their orthologs that have a function in plant Fe deficiency, such as *CgbHLH16*, *CgbHLH48*, *CgbHLH62*, *CgbHLH63*, *CgbHLH79*, *CgbHLH80*, *CgbHLH123*, *CgbHLH128* and *CgbHLH130.2*. New and essential bHLHs that regulate Fe homeostasis are expected to be identified from these candidates in future. In particular, *CgbHLH16* was continuously up-regulated at all tested times of –Fe treatment, and it was also predicted to interact with an important Fe deficiency-responsive TF, PYE (bHLH47), based on protein-protein interaction prediction (Fig. 6). In addition, *CgbHLH62* and *CgbHLH63* were predicted to interact with each other while their expression levels showed the opposite tendency at the same time points, which indicates that there may be negative regulation relationships between them under Fe deficiency. Overall, these results provide us abundant information for understanding of the citrus *bHLH* genes, but more work including transgenic and biochemical experiments needs to be done to further confirm our speculations and illustrate their functions.

## Conclusions

In summary, 128 CgbHLH proteins were identified from pummelo (*Citrus grandis*) genome and were classified into 18 subfamilies based on the phylogenetic relationship with AtbHLH proteins. The detailed analysis of Mw, pI, sequence alignment, phylogeny, gene duplication, chromosomal distribution, gene structure and protein motif suggested basic properties of each CgbHLH. GO annotation and protein-protein interaction analysis further revealed the potential functions of *CgbHLHs*. qRT-PCR showed that 34 of the tested *CgbHLH* genes were up- or down-regulated under Fe deficiency, and some of them such as *CgbHLH6*, *CgbHLH14.2*, *CgbHLH16*, *CgbHLH48*, *CgbHLH63*, *CgbHLH79*, *CgbHLH80*, *CgbHLH104*, *CgbHLH105.1*, *CgbHLH123*, *CgbHLH128*, *CgbHLH93.1*, *CgbHLH62*, *CgbHLH130.2*, *CgbHLH3*, *CgbHLH29.4*, *CgbHLH14.1*, *CgbHLH69.1*, *CgbHLH153* and *CgbHLH77* were considered the key candidates that respond to Fe deficiency. This study lays the foundation for further functional elucidation of *CgbHLH* genes in citrus.

## Methods

### Plant material and treatment

Seeds of pummelo (*Citrus grandis*) were collected from the Citrus Research Institute, Southwest University, Chongqing, China. After surface disinfection with 2% sodium hypochlorite, seed coats were removed, and seeds were then germinated on wet filter papers that filled 96-cell plastic boxes with lids. After 2 weeks germination, uniform seedlings were transferred into modified Hoagland’s solution for hydroponics as described by Fu et al. (2017) [1]. When the seedlings had grown four leaves, half of them were renewed with Fe deficient (0.5 μM Fe-EDTA) solution (−Fe), while the others received normal (50 μM Fe-EDTA) solution (CK). The culture conditions were a temperature of 28 °C and a relative humidity of 70% under a 16-h photoperiod (50 μmol m^− 2^ s^− 1^). Both –Fe and CK groups had three biological replicates, and each replicate contained 14 seedlings. After 0.5 d, 1.5 d, 2 d, 7 d, and 12 d of treatment, roots of –Fe and CK were sampled and then frozen in liquid nitrogen immediately.

### Identification of bHLH members in pummelo

The genome data of pummelo were downloaded from the Orange (*Citrus sinensis*) Genome Annotation Project (http://citrus.hzau.edu.cn/orange/download/index.php). Then, we used two methods to search for all bHLHs of pummelo. Firstly, the hidden Markov model (HMM) file of the HLH domain (PF00010) obtained from the Pfam database (http://pfam.xfam.org/) was used as the query to scan the pummelo genome with HMMER software (http://hmmer.org/). Secondly, the bHLH domain sequences of *A. thaliana* (AtbHLHs) were used as queries to blast against the pummelo genome. After removal of redundant sequences, all putative bHLHs obtained from the two methods were submitted to the NCBI (National Center of Biotechnology Information) conserved domain database (CDD, https://www.ncbi.nlm.nih.gov/Structure/bwrpsb/bwrpsb.cgi) to further verify the existence of the conserved bHLH domain. All the identified bHLH proteins of pummelo were named as CgbHLHs.

### Multiple sequence alignment and phylogenetic tree construction

Molecular weights (MWs) and isoelectric points (pIs) of CgbHLH proteins were analyzed with ExPASy (http://web.expasy.org/compute_pi/). Conserved domain sequences of CgbHLHs were submitted to ClustalW to perform multiple sequence alignment, and the BioEdit was used to edit the aligned sequences and shade the conserved sites. To visualize the conserved motifs, the sequences were analyzed with WEBLOGO programs (http://weblogo.berkeley.edu). The neighbor-joining phylogenetic tree was constructed by using MEGA7 and 1000 replicates in bootstrap analysis. In addition, the full-length protein sequences of all CgbHLHs and known AtbHLHs were also aligned in Clustal Omega (https://www.ebi.ac.uk/Tools/msa/clustalo/) to obtain their sequence identity (%) data, and the CgbHLH-AtbHLH pairs with highest percent identity were considered orthologs.

### Gene structure, conserved motifs, chromosomal location and gene duplication analysis

Gene structure information of each CgbHLH was obtained from the generic feature format (GFF) file downloaded from the pummelo genome database, and TBtools [60] was used to display their intron-exon structures. Conserved motifs of each CgbHLH protein were predicted in the MEME [61] web tool with the maximum number of motifs set at 20, and the other parameters were the default values. The chromosomal locations of the CgbHLH genes were provided by the pummelo genome database, and TBtools software was used to draw the location images. To predict the gene duplication, the BLASTP program was first used to blast against the pummelo genome using all peptide sequences of pummelo as the queries; then, the top matches (e value ≤1e− 05) and their GFF files were inputted into MCScanX software [62] to identify tandem duplications, segmental duplications, and collinear correlations.

### Gene ontology (GO) annotation and RNA-sequencing (RNAseq) data analysis

GO annotation of all the CgbHLH proteins was performed with the Blast2GO program, and *A. thaliana* was chosen as the reference. To evaluate the expression levels of CgbHLH genes in pummelo, the published RNAseq raw data (SRR4050288/SRR4050289) of pummelo [39] were downloaded from the NCBI database (https://www.ncbi.nlm.nih.gov/sra/). Then, the raw data were analyzed with the linux system of the fastqc and trimommatic programs to remove adaptors and low quality sequences. The kallisto program was used to map sequence data to the pummelo genome, and the featurecount program was used to calculate the values of the transcripts per million reads (TPM).

### Prediction of the protein-protein interaction network

The interaction network of CgbHLHs was predicted in STRING (version 11.0, http://string-db.org) website that contains known and predicted protein-protein interactions of different organisms [63]. In detail, all the CgbHLH protein sequences were first submitted to the STRING website as queries, and *A. thaliana* was chosen as the reference organism for blasting because pummulo has no protein-protein interaction database and is not included in STRING. After blasting, the matched homologs of *A. thaliana* with the highest scores (Bitscore) and higher than 40% identity were used to construct the network. To ensure reliability, only the predicted interactions with interaction scores > 0.7 (high confidence level was set in STRING) were shown in the network.

### RNA extraction and quantitative real-time PCR (qRT-PCR) analysis

Total RNA was extracted from the –Fe and CK roots of pummelo by using the RNAprep pure plant kit (Cat#DP432, Tiangen Biotech Co., Ltd., Beijing, China), and the RNA concentration and quality were determined with a Nanodrop 2000 spectrophotometer (Thermo Scientific, Waltham, MA, USA). Then, 1 μg of high-quality RNA was used for cDNA synthesis with HiScript II Q RT Super Mix (Cat#R222–01, Vazyme Biotech Co., Ltd., Nanjing, China) according to the manufacturer’s instructions. qRT-PCR was performed on the Bio-Rad CFX Connect RealTime system by using ChamQ™ Universal SYBR® qPCR Master Mix (Cat#Q711, Vazyme Biotech Co., Ltd., Nanjing, China). Each PCR reaction contained 5.0 μL SYBR mix, 0.2 μM primers, and 1.0 μL diluted cDNA in a final volume of 10 μL. The 2^-ΔΔCT^ method was used to normalize and calculate the expression level of each tested gene relative to an internal reference gene, actin (Cs1g05000.1). All the primers of *CgbHLH* genes are listed in Additional file 3: Table S2. Three biological replicates and three technical replicates were performed for each treatment. The presented data herein are means ± standard errors (SE) of three biological replicates. Student’s *t*-test was used to analyze the statistical differences between -Fe and corresponding CK samples, taking *P* < 0.05 as significantly different.

## Supplementary information

**Additional file 1: Figure S1.** Sequence alignment of 128 CgbHLH proteins.

**Additional file 2: Table S1.** Predicted MW, pI, exon number, UTR and motifs of 128 CgbHLH proteins.

**Additional file 3: Table S2.** The primers of *CgbHLH* genes used for qRT-PCR.

## Data Availability

All data generated during this study are included in this published article and its supplementary information files. In addition, the protein and CDS sequences of CgbHLHs analysed in this study were retrieved from the Orange Genome Annotation Project, http://citrus.hzau.edu.cn/orange/download/index.php. The protein sequences of AtbHLHs were retrieved from the TAIR database, https://www.arabidopsis.org/browse/genefamily/bHLH.jsp.

## Abbreviations

ABS5: Abnormal shoot 5
AKS2: ABA-responsive kinase substrate 2
ALC: ALCATRAZ
AMS: Aborted microspores
BEE1: BR enhanced expression 1
bHLH: Basic/helix-loop-helix
BIM1: BES1-interacting myc-like 1
BP: Biological process
CC: Cellular component
CDD: Conserved domain database
CES: CESTA
CIB1: Cryptochrome-interacting basic/helix-loop-helix 1
CIL2: CIB1 like protein 2
CITF1: Cu-deficiency induced transcription factor 1
DYT1: Dysfunctional tapetum 1
EGL3: Enhancer of glabra 3
FBH1: Flowering bHLH 1
Fe: Iron
FIT: FER-like iron-deficiency-induced transcription factor
FRO2: Ferric reduction oxidase 2
FRU: Fer-like iron deficiency induced transcription factor 3
GO: Gene ontology
HEC: Hecate
HMM: Hidden markov model
ICE2: Inducer of CBF expression 2
ILR3: IAA-leucine resistant 3
IRT1: Iron-regulated transporter 1
JA: Jasmonic acid
JAI1: Jasmonate insensitive 1
JAM: Jasmonate associated MYC2 like
LHL2: Lonesome highway like 2
LRL2: LJRHL1-like 2
MF: Molecular function
MW: Molecular weight
NCBI: National center of biotechnology information
NFL: No flowering in short day
ORG3: OBP3-responsive gene 3
pI: Isoelectric point
PIF: Phytochrome interacting factor
PIL5: Phytochrome interacting factor 3-like 5
PYE: POPEYE
qRT-PCR: Quantitative real-time PCR
RGE1: Retarded growth of embryo 1
RHD6: Root hair defective 6
ROS: Reactive oxygen species
RSL2: Root hair defective 6-like 2
SPCH: Speechless
SPT: SPATULA
TFs: Transcription factors
TMO5: Target of monopteros 5
TPM: Transcripts per million reads
TT8: Transparent testa 8
UNE10: Unfertilized embryo sac 10
UTR: Untranslated region
WGD: Whole-genome duplication event
