# Supplementary figures and images for: Systematic analysis of the basic/helix-loop-helix (bHLH) transcription factor family in pummelo (Citrus grandis) and identification of the key members involved in the response to iron deficiency
Source: BMC Genomics. 2020 Mar 14;21:233. doi: 10.1186/s12864-020-6644-7 (PMC7071715; doi:10.1186/s12864-020-6644-7)

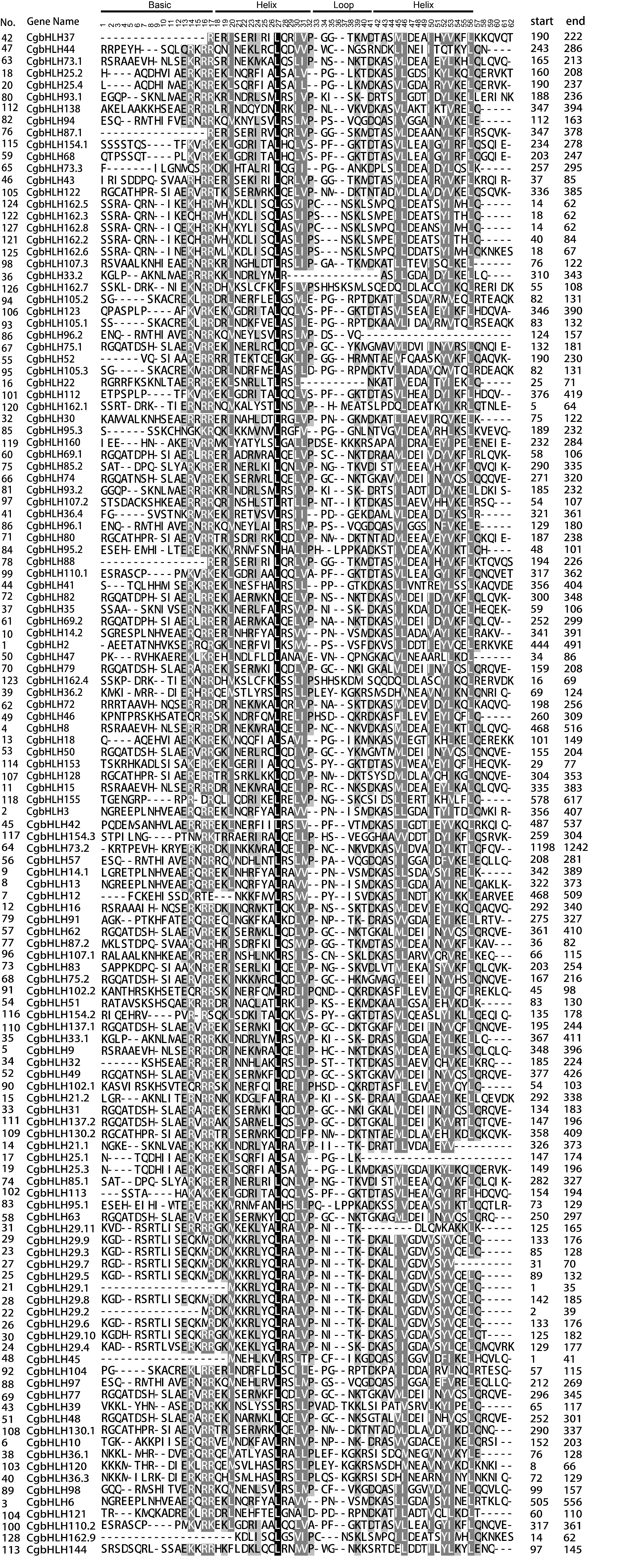

Supplement: Supplementary file 1 — Additional file 1: Figure S1. Sequence alignment of 128 CgbHLH proteins. [file 12864_2020_6644_MOESM1_ESM.jpg]
