# Supplementary material for: Systematic analysis of the basic/helix-loop-helix (bHLH) transcription factor family in pummelo (Citrus grandis) and identification of the key members involved in the response to iron deficiency
Source: BMC Genomics. 2020 Mar 14;21:233. doi: 10.1186/s12864-020-6644-7 (PMC7071715; doi:10.1186/s12864-020-6644-7)
Supplement: Supplementary file 2 — Additional file 2: Table S1. Predicted MW, pI, exon number, UTR and motifs of 128 CgbHLH proteins. [file 12864_2020_6644_MOESM2_ESM.docx]

Table S1 Predicted MW, pI, exon number, UTR and motifs of 128 CgbHLH proteins.

| **Subfamily** | **No.** | **Gene Name** | **Gene ID** | **Mw**  **(kDa)** | **pI** | **No.**  **exon** | **UTR** | **Predicted motifs** |
| --- | --- | --- | --- | --- | --- | --- | --- | --- |
| **(1)** | 14 | CgbHLH21.1 | Cg8g001500 | 66.60 | 4.91 | 8 | Non | 1, 2, 3, 4, 12, 14, 15 |
|  | 15 | CgbHLH21.2 | Cg7g019250 | 53.03 | 6.20 | 8 | Yes | 1, 2, 3, 4, 12, 14, 20 |
|  | 21 | CgbHLH29.1 | Cg8g019060 | 7.56 | 9.17 | 2 | Non | 1, 2, 8 |
|  | 22 | CgbHLH29.2 | Cg8g019160 | 8.09 | 9.37 | 2 | Non | 1, 2, 8 |
|  | 23 | CgbHLH29.3 | Cg8g018990 | 17.75 | 5.15 | 3 | Non | 1, 2, 3, 5, 8 |
|  | 24 | CgbHLH29.4 | Cg8g019240 | 35.90 | 5.01 | 4 | Non | 1, 2, 3, 5, 8 |
|  | 25 | CgbHLH29.5 | Cg8g019040 | 18.21 | 5.78 | 3 | Non | 1, 2, 3, 5, 8 |
|  | 26 | CgbHLH29.6 | Cg8g019200 | 23.23 | 4.79 | 2 | Non | 1, 2, 3, 5, 8 |
|  | 27 | CgbHLH29.7 | Cg8g019020 | 8.99 | 10.41 | 2 | Non | 1, 3, 19 |
|  | 28 | CgbHLH29.8 | Cg8g019140 | 24.67 | 8.97 | 4 | Non | 1, 2, 3, 5, 8, 16 |
|  | 29 | CgbHLH29.9 | Cg8g018940 | 24.34 | 8.47 | 5 | Non | 1, 2, 3, 5, 8, 16 |
|  | 30 | CgbHLH29.10 | Cg8g019230 | 52.50 | 4.94 | 6 | Non | 1, 2, 3, 4, 5, 8 |
|  | 31 | CgbHLH29.11 | Cg8g018890 | 33.17 | 6.05 | 4 | Non | 1, 3, 5, 8 |
|  | 35 | CgbHLH33.1 | Cg7g011080 | 60.39 | 5.21 | 4 | Yes | 1, 2, 3, 4, 5, 6 |
|  | 36 | CgbHLH33.2 | Cg2g009820 | 52.81 | 5.23 | 4 | Yes | 2, 3, 4, 5 |
|  | 80 | CgbHLH93.1 | Cg1g007400 | 39.51 | 5.10 | 4 | Yes | 1, 2, 3, 4, 15 |
|  | 81 | CgbHLH93.2 | Cg3g013530 | 43.54 | 4.71 | 4 | Non | 1, 2, 3, 4, 15 |
| **(2)** | 37 | CgbHLH35 | Cg4g021000 | 28.22 | 5.10 | 5 | Non | 1, 2, 3, 4, 16 |
|  | 48 | CgbHLH45 | Cg9g004620 | 20.36 | 6.96 | 3 | Non | 2, 4, 7, 15 |
|  | 56 | CgbHLH57 | Cg5g039930 | 48.80 | 5.63 | 4 | Yes | 2, 3, 3, 4, 7, 8, 15, 19 |
|  | 82 | CgbHLH94 | Cg1g008260 | 35.59 | 7.71 | 4 | Non | 2, 3, 4, 6, 7, 15 |
|  | 86 | CgbHLH96.1 | Cg3g014510 | 36.42 | 5.58 | 3 | Yes | 2, 3, 4, 7, 15, 20 |
|  | 87 | CgbHLH96.2 | Cg2g030740 | 34.12 | 5.10 | 3 | Yes | 3, 4, 7, 15 |
|  | 88 | CgbHLH97 | Cg9g012540 | 48.38 | 5.38 | 4 | Yes | 2, 3, 4, 7, 15, 19 |
|  | 89 | CgbHLH98 | Cg9g028240 | 34.93 | 5.59 | 2 | Non | 1, 2, 3, 4, 7, 8, 15 |
| **(3)** | 6 | CgbHLH10 | Cg9g023740 | 39.79 | 5.71 | 3 | Non | 2, 3, 4, 7, 15 |
|  | 16 | CgbHLH22 | Cg2g040910 | 22.11 | 8.37 | 4 | Non | 2, 3, 4 |
|  | 79 | CgbHLH91 | Cg5g045050 | 53.35 | 5.71 | 4 | Yes | 1, 2, 3, 3, 4, 15 |
| **(4)** | 38 | CgbHLH36.1 | Cg9g025850 | 27.71 | 6.32 | 3 | Non | 2, 3, 4, 7 |
|  | 39 | CgbHLH36.2 | Cg5g009400 | 28.25 | 6.32 | 3 | Yes | 2, 3, 4, 7, 19 |
|  | 40 | CgbHLH36.3 | Cg9g025870 | 27.19 | 8.29 | 3 | Yes | 2, 3, 4, 7, 19 |
|  | 43 | CgbHLH39 | Cg9g016290 | 28.75 | 6.33 | 3 | Non | 2, 3, 4, 7 |
|  | 103 | CgbHLH120 | Cg9g025860 | 19.66 | 6.96 | 3 | Non | 2, 3, 4, 7 |
|  | 119 | CgbHLH160 | Cg3g004340 | 37.22 | 4.61 | 3 | Non | 2, 3, 7 |
| **(5)** | 83 | CgbHLH95.1 | Cg8g004350 | 34.17 | 8.84 | 3 | Non | 2, 3, 4, 7, 15 |
|  | 84 | CgbHLH95.2 | Cg3g021160 | 29.80 | 6.31 | 3 | Non | 2, 3, 4, 7, 15 |
|  | 120 | CgbHLH162.1 | Cg2g042210 | 21.06 | 6.09 | 4 | Yes | 2, 3, 4, 7, 8, 9 |
|  | 121 | CgbHLH162.2 | Cg2g005390 | 12.84 | 7.85 | 3 | Yes | 2, 3, 7 |
|  | 122 | CgbHLH162.3 | Cg2g005350 | 10.36 | 9.22 | 3 | Non | 2, 3, 7 |
|  | 123 | CgbHLH162.4 | Cg5g004810 | 20.66 | 9.05 | 3 | Yes | 2, 3, 4, 7, 9 |
|  | 124 | CgbHLH162.5 | Cg2g005320 | 18.39 | 7.77 | 3 | Non | 2, 3, 4, 7, 9 |
|  | 125 | CgbHLH162.6 | Cg2g005400 | 16.91 | 7.94 | 2 | Non | 2, 3, 4, 7, 9 |
|  | 126 | CgbHLH162.7 | Cg2g015830 | 25.40 | 9.11 | 3 | Yes | 2, 3, 4, 7, 9 |
|  | 127 | CgbHLH162.8 | Cg2g005380 | 18.43 | 9.22 | 3 | Non | 2, 3, 4, 7, 9 |
|  | 128 | CgbHLH162.9 | Cg2g005340 | 18.39 | 7.77 | 3 | Non | 2, 3, 4, 7, 9 |
| **(6)** | 50 | CgbHLH47 | Cg5g003170 | 26.57 | 5.33 | 5 | Yes | 2, 3, 15, 16 |
|  | 92 | CgbHLH104 | Cg9g007860 | 24.50 | 5.69 | 5 | Yes | 2, 3, 10, 16 |
|  | 93 | CgbHLH105.1 | Cg2g023210 | 25.96 | 8.36 | 5 | Yes | 2, 3, 7, 10, 16 |
|  | 94 | CgbHLH105.2 | Cg2g017780 | 25.82 | 5.61 | 6 | Yes | 1, 2, 3, 10, 16 |
|  | 95 | CgbHLH105.3 | Cg2g040740 | 25.83 | 7.63 | 5 | Yes | 2, 3, 7, 10, 16 |
|  | 104 | CgbHLH121 | CgUng005820 | 37.64 | 6.93 | 6 | Yes | 2, 3, 7, 15, 16 |
| **(7)** | 1 | CgbHLH2 | Cg5g002660 | 69.64 | 5.60 | 7 | Yes | 1, 2, 3, 4, 11, 12, 14, 15, 17, 18 |
|  | 7 | CgbHLH12 | Cg5g042050 | 73.36 | 5.14 | 8 | Yes | 1, 2, 4, 11, 12, 14, 15, 17, 18 |
|  | 44 | CgbHLH41 | Cg4g008800 | 59.06 | 6.50 | 8 | Yes | 1, 2, 3, 12, 18, 18, 19 |
|  | 45 | CgbHLH42 | Cg5g035630 | 76.27 | 4.89 | 14 | Yes | 1, 2, 3, 4, 11, 12, 14, 15, 17, 18 |
| **(8)** | 13 | CgbHLH18 | Cg5g017710 | 29.21 | 9.14 | 3 | Non | 1, 2, 3, 4, 15 |
|  | 17 | CgbHLH25.1 | Cg8g002160 | 21.01 | 9.13 | 3 | Yes | 1, 3, 20 |
|  | 18 | CgbHLH25.2 | Cg1g007240 | 37.65 | 5.85 | 4 | Non | 1, 2, 3, 4, 15, 20 |
|  | 19 | CgbHLH25.3 | Cg8g002170 | 15.95 | 5.94 | 2 | Yes | 1, 2, 3, 4, 15, 20 |
|  | 20 | CgbHLH25.4 | Cg1g007260 | 40.86 | 8.41 | 4 | Non | 1, 2, 3, 4, 15 |
| **(9)** | 33 | CgbHLH31 | Cg7g020320 | 27.71 | 6.46 | 6 | Yes | 1, 2, 3, 6 |
|  | 51 | CgbHLH48 | Cg9g022350 | 45.05 | 6.49 | 7 | Yes | 1, 2, 3, 3, 6, 19 |
|  | 52 | CgbHLH49 | Cg7g015600 | 59.48 | 5.72 | 10 | Yes | 1, 2, 3, 6, 8, 13 |
|  | 53 | CgbHLH50 | Cg5g022610 | 30.10 | 6.85 | 6 | Non | 1, 2, 3, 3, 6 |
|  | 57 | CgbHLH62 | Cg6g008950 | 60.06 | 6.12 | 9 | Yes | 1, 2, 3, 6 |
|  | 58 | CgbHLH63 | Cg8g007040 | 44.81 | 7.02 | 7 | Yes | 1, 2, 3, 6, 19 |
|  | 60 | CgbHLH69.1 | Cg3g010300 | 22.90 | 9.06 | 6 | Yes | 1, 2, 3, 6, 13, 19 |
|  | 61 | CgbHLH69.2 | Cg4g024560 | 37.19 | 5.90 | 7 | Yes | 1, 2, 3, 6, 19, 19 |
|  | 66 | CgbHLH74 | Cg3g013130 | 46.43 | 5.44 | 9 | Yes | 1, 2, 3, 6 |
|  | 67 | CgbHLH75.1 | Cg2g036150 | 26.42 | 6.39 | 6 | Yes | 1, 2, 3, 6, 8, 19 |
|  | 68 | CgbHLH75.2 | Cg6g019500 | 41.49 | 8.83 | 6 | Yes | 1, 2, 3, 6, 6, 6 |
|  | 69 | CgbHLH77 | Cg9g014180 | 52.72 | 6.21 | 8 | Yes | 1, 2, 3, 6 |
|  | 70 | CgbHLH79 | Cg5g004140 | 30.06 | 5.77 | 6 | Yes | 1, 2, 3, 6, 19 |
|  | 72 | CgbHLH82 | Cg4g018170 | 56.29 | 5.00 | 8 | Yes | 1, 2, 3, 6, 13 |
|  | 110 | CgbHLH137.1 | Cg7g010980 | 41.60 | 6.42 | 8 | Yes | 1, 2, 3, 3, 6, 16 |
|  | 111 | CgbHLH137.2 | Cg7g022310 | 31.16 | 7.75 | 7 | Non | 1, 2, 3, 3, 6, 16 |
|  | 117 | CgbHLH154.3 | Cg5g035790 | 41.21 | 4.98 | 8 | Yes | 1, 2 |
| **(10)** | 4 | CgbHLH8 | Cg5g013200 | 79.45 | 5.89 | 9 | Yes | 1, 2, 3, 8 |
|  | 5 | CgbHLH9 | Cg7g012190 | 59.63 | 7.22 | 9 | Yes | 1, 2, 3, 7 |
|  | 11 | CgbHLH15 | Cg5g032930 | 64.26 | 7.69 | 7 | Yes | 1, 2, 3, 6, 19, 19 |
|  | 12 | CgbHLH16 | Cg5g043840 | 50.31 | 8.82 | 6 | Yes | 1, 2, 3, 19, 19 |
|  | 62 | CgbHLH72 | Cg5g010830 | 42.09 | 9.34 | 6 | Non | 1, 2, 3, 19, 19 |
|  | 63 | CgbHLH73.1 | Cg1g005520 | 44.20 | 6.33 | 8 | Yes | 1, 2, 3 |
|  | 64 | CgbHLH73.2 | Cg5g039380 | 163.79 | 9.09 | 21 | Non | 1, 2, 3, 10, 15, 16 |
| **(11)** | 42 | CgbHLH37 | Cg1g002590 | 29.77 | 9.24 | 1 | Non | 1, 2, 3 |
|  | 46 | CgbHLH43 | Cg1g025450 | 17.12 | 10.13 | 1 | Non | 1, 2, 3 |
|  | 76 | CgbHLH87.1 | Cg1g009170 | 47.56 | 6.02 | 2 | Non | 1, 2, 3, 5, 8 |
|  | 77 | CgbHLH87.2 | Cg6g009450 | 18.99 | 7.11 | 1 | Non | 1, 2, 3 |
|  | 78 | CgbHLH88 | Cg3g021250 | 29.97 | 8.61 | 1 | Yes | 1, 2, 3 |
| **(12)** | 55 | CgbHLH52 | Cg2g038660 | 35.31 | 5.38 | 2 | Non | 1, 2, 3, 13, 19, 19 |
|  | 73 | CgbHLH83 | Cg6g018550 | 32.51 | 6.33 | 5 | Non | 1, 2, 3 |
|  | 74 | CgbHLH85.1 | Cg8g003130 | 39.59 | 4.91 | 4 | Non | 1, 2, 3, 6, 8 |
|  | 75 | CgbHLH85.2 | Cg3g012730 | 40.59 | 5.28 | 5 | Non | 1, 2, 3, 6, 19 |
| **(13)** | 47 | CgbHLH44 | Cg1g004400 | 45.02 | 7.07 | 5 | Non | 1, 2, 3 |
|  | 71 | CgbHLH80 | Cg3g020720 | 27.67 | 6.13 | 5 | Yes | 1, 2, 3, 6 |
|  | 105 | CgbHLH122 | Cg2g002540 | 48.30 | 6.49 | 6 | Yes | 1, 2, 3, 5, 6, 19, 19, 19 |
|  | 107 | CgbHLH128 | Cg5g028960 | 39.18 | 8.44 | 6 | Yes | 1, 2, 3, 6, 8 |
|  | 108 | CgbHLH130.1 | Cg9g022410 | 85.38 | 9.54 | 11 | Non | 1, 2, 3, 6, 6, 11 |
|  | 109 | CgbHLH130.2 | Cg7g023040 | 46.91 | 7.53 | 8 | Yes | 1, 2, 3, 6 |
|  | 118 | CgbHLH155 | Cg5g034200 | 82.48 | 5.67 | 12 | Yes | 1, 1, 1, 2, 4, 12, 14 |
| **(14)** | 59 | CgbHLH68 | Cg1g014350 | 36.87 | 6.42 | 9 | Yes | 1, 2, 13 |
|  | 99 | CgbHLH110.1 | Cg3g023160 | 46.48 | 7.66 | 7 | Yes | 1, 2, 13 |
|  | 100 | CgbHLH110.2 | CgUng019530 | 39.82 | 6.63 | 5 | Yes | 1, 2 |
|  | 101 | CgbHLH112 | Cg2g040950 | 53.33 | 6.14 | 8 | Yes | 1, 2, 13, 19 |
|  | 102 | CgbHLH113 | Cg8g003580 | 28.46 | 7.62 | 4 | Yes | 1, 2, 13 |
|  | 106 | CgbHLH123 | Cg2g022720 | 49.65 | 6.81 | 7 | Yes | 1, 2, 13, 19, 19 |
|  | 114 | CgbHLH153 | Cg5g026370 | 15.95 | 9.19 | 7 | Yes | 1, 2, 3, 13 |
|  | 115 | CgbHLH154.1 | Cg1g012580 | 33.90 | 8.82 | 8 | Yes | 1, 2 |
|  | 116 | CgbHLH154.2 | Cg7g004740 | 27.52 | 6.96 | 7 | Non | 1, 2, 13 |
| **(15)** | 41 | CgbHLH36.4 | Cg3g014280 | 40.74 | 4.55 | 4 | Yes | 2, 7, 14 |
|  | 65 | CgbHLH73.3 | Cg1g019630 | 33.10 | 5.33 | 2 | Non | 1, 2 |
|  | 85 | CgbHLH95.3 | Cg2g044520 | 26.84 | 5.51 | 3 | Yes | 1, 2 |
|  | 113 | CgbHLH144 | Cg1g004390 | 22.50 | 9.14 | 3 | Non | 1, 2, 3, 19 |
| **(16)** | 49 | CgbHLH46 | Cg5g012000 | 64.08 | 8.96 | 13 | Yes | 1, 2, 3, 4 |
|  | 90 | CgbHLH102.1 | Cg7g015780 | 38.18 | 6.35 | 8 | Yes | 1, 1, 2, 3, 4 |
|  | 91 | CgbHLH102.2 | Cg6g020770 | 37.95 | 5.53 | 7 | Yes | 1, 2, 3, 4, 19 |
| **(17)** | 32 | CgbHLH30 | Cg2g043260 | 28.86 | 6.77 | 2 | Yes | 1, 2, 3, 4 |
|  | 34 | CgbHLH32 | Cg7g014890 | 29.43 | 6.20 | 3 | Yes | 1, 2, 3, 19, 19 |
|  | 54 | CgbHLH51 | Cg7g002190 | 31.02 | 8.92 | 2 | Yes | 1, 2, 3, 4, 6 |
|  | 96 | CgbHLH107.1 | Cg6g017840 | 26.80 | 7.01 | 2 | Yes | 1, 2, 3, 4 |
|  | 97 | CgbHLH107.2 | Cg3g014080 | 28.92 | 7.09 | 2 | Yes | 1, 2, 3, 4 |
|  | 98 | CgbHLH107.3 | Cg2g008600 | 31.29 | 5.94 | 3 | Yes | 1, 2, 3, 4 |
|  | 112 | CgbHLH138 | Cg1g007950 | 57.85 | 9.20 | 4 | Non | 1, 2, 3, 4 |
| **(18)** | 2 | CgbHLH3 | Cg5g034370 | 55.73 | 6.16 | 2 | Yes | 1, 2, 3, 4, 11, 12, 12, 14, 15, 18 |
|  | 3 | CgbHLH6 | CgUng000770 | 74.76 | 5.50 | 1 | Yes | 1, 2, 3, 3, 3, 4, 11, 12, 14, 15, 18, 19, 19 |
|  | 8 | CgbHLH13 | Cg5g040610 | 54.56 | 8.18 | 2 | Yes | 1, 2, 3, 4, 11, 12, 15, 18 |
|  | 9 | CgbHLH14.1 | Cg5g040200 | 57.24 | 5.57 | 1 | Yes | 1, 2, 3, 4, 11, 12, 14, 15, 17, 18 |
|  | 10 | CgbHLH14.2 | Cg5g000450 | 58.22 | 6.33 | 2 | Yes | 1, 2, 3, 4, 11, 12, 14, 15, 18 |

Mw, molecular weight, kDa; pI, isoelectric point; UTR, untranslated region. The Gene ID derived from the Pummelo genome database (<http://citrus.hzau.edu.cn/orange/index.php>). The sequences and length of 1 to 20 motifs are as follows.

Motif 1: JNERLRALQALVPNITKMDKA (21 aa)

Motif 2: SILDEAIEYVKELQKQVKELS (21 aa)

Motif 3: ATDSHSJAERKRREK (15 aa)

Motif 4: LLKIISALEELGLEVVNANVSTVGDRVLYSFVVKVKDG (38 aa)

Motif 5: NFILNSSTLSSLNLDGEVKEGDNCHEDKDGDDASGATRTTTIIDASSKKP (60 aa)

Motif 6: DYIHVRARRGQ (11 aa)

Motif 7: MKDLLSVLRSLIPSNYVKRMP (21 aa)

Motif 8: TEVAGCEASPSSSENSKESIE (21 aa)

Motif 9: HPEPPVMNISTSGSTLEVNLICGLNRNFM (29 aa)

Motif 10: AAFAAQGQVAGNKLVPFIGYPGVAMWQFMPPAAVDTSQDHVLRPPVA (47 aa)

Motif 11: VTDTEWFYLVSMSFSFPIGEG (21 aa)

Motif 12: QTVVCIPTLDGVVELGSTELI (21 aa)

Motif 13: KQDLRSRGLCLVPISCTSAVA (21 aa)

Motif 14: LRLQLAVAVRSEQWSYAIFWQ (21 aa)

Motif 15: DVEVKISGSDALIKILSPKKPGQ (23 aa)

Motif 16: IKELKAEKNELRDEKQRLKAEKENJERQI (29 aa)

Motif 17: QGVLEWGDGYYNGDIKTRKTIQPMELSPDQJGLQRSQQLRELYESLSVGE (50 aa)

Motif 18: GRAYASGQHVWLTGAQELDSKVCSRALLA (29 aa)

Motif 19: QQQQQQQQQQQQQ (13 aa)

Motif 20: WHMLNSIDEFNLLPIAAAFGENLQHSYTHPSFNNNTGIDRPQKQLKTSTW (50 aa)
