# Supplementary material for: Systematic analysis of the basic/helix-loop-helix (bHLH) transcription factor family in pummelo (Citrus grandis) and identification of the key members involved in the response to iron deficiency
Source: BMC Genomics. 2020 Mar 14;21:233. doi: 10.1186/s12864-020-6644-7 (PMC7071715; doi:10.1186/s12864-020-6644-7)
Supplement: Supplementary file 3 — Additional file 3: Table S2. The primers of CgbHLH genes used for qRT-PCR. [file 12864_2020_6644_MOESM3_ESM.docx]

| **No.** | **Gene Name** | **Primer** | **No.** | **Gene Name** | **Primer** |
| --- | --- | --- | --- | --- | --- |
| **2** | *CgbHLH3* | F: GTGTGCACCAGAGGCTATGT | **63** | *CgbHLH73.1* | F: GGACATGTTGCCACACCAAC |
|  |  | R: CCATCCTTTCGGTCACAGCA |  |  | R: CACCTTCTGCACGGTCTCTT |
| **3** | *CgbHLH6* | F: TTCTGCATCTACAGCCGACC | **69** | *CgbHLH77* | F: CAAGAGCAGCAACAATTTGCC |
|  |  | R: CCGGTGTAATCGCATGAGGA |  |  | R: CTATCAGAAGCAGAAGCAGAAACAG |
| **8** | *CgbHLH13* | F: CTGCCCATGATGAGGTGGTT | **70** | *CgbHLH79* | F: GTTCGGCTCTCAGAGATGGG |
|  |  | R: TTGTCAACTGCTCCGATCCC |  |  | R: TCTCTTTCTGCCACACCCAC |
| **9** | *CgbHLH14.1* | F: GGTCAAGAAACCACGCAAAG | **71** | *CgbHLH80* | F: AGTGCCTTGTAGGGTTCGTG |
|  |  | R: TTGGCGTTCTTTTCTCCATAGT |  |  | R: TGTCCATGTTCGGCACAAGA |
| **10** | *CgbHLH14.2* | F: CTATGCTCTCCGCTCTGTGG | **79** | *CgbHLH91* | F: GGTTCTTGACGAGCTTCGGT |
|  |  | R: GGCCCTGAGCTCTTTGATGT |  |  | R: CTAGTTGGTGGAACTGCTGC |
| **12** | *CgbHLH16* | F: GCAACGGACCAGTTCCAGAT | **80** | *CgbHLH93.1* | F: GAGAGAGATCGACACCCGGA |
|  |  | R: GTCTCTGCTGCTCCACTCTG |  |  | R: GCTCAGCTGCCTCTGAACAT |
| **18** | *CgbHLH25.2* | F: GATGACCTCGTCTCCTGCAC | **81** | *CgbHLH93.2* | F: TCTTGCTCAGAGGGCAATGT |
|  |  | R: GCTCTTGCACCTGAGCAATC |  |  | R: TGCTCATTGGCTGTCCTGTT |
| **24** | *CgbHLH29.4* | F: AGATTGGTAAGCAGCGGAGG | **86** | *CgbHLH96.1* | F: ACGACCTAGGCAGCTCTTGA |
|  |  | R: GGCAATTGGTCATCAGTTACCAG |  |  | R: TGCTTCCTCCTCAATTTCCAGT |
| **30** | *CgbHLH29.10* | F: ATTTGAAGCTGTGGGTGGCT | **90** | *CgbHLH102.1* | F: CGAAAGGAAATCGAGAGGAGGA |
|  |  | R: AACCACATCCAACGGCTGAT |  |  | R: ACACTGGCCTTATCGCTGT |
| **32** | *CgbHLH30* | F: GGCATCTATCTGCTGCGAGT | **92** | *CgbHLH104* | F: CCGTCAGACTGGGACGATTT |
|  |  | R: AGAGTTTGGGATGCCTCAGC |  |  | R: TTTTCTGGCGGAACAATCCC |
| **33** | *CgbHLH31* | F: GAATCTGGAGGTGGGTTGGG | **93** | *CgbHLH105.1* | F: TCGTGATGAGAAGCAGAGGC |
|  |  | R: CCATCAGAATCATTATGCTTGGCT |  |  | R: CTTGTTTCCAGGTGCTTGGC |
| **35** | *CgbHLH33.1* | F: GCCAACTTTTGAGTGGGATTGA | **94** | *CgbHLH105.2* | F: GCCCAGAAACTGAAGCAATCA |
|  |  | R: TTGAGACCCGACCCATCAAC |  |  | R: GCAAATGCAGCCGACATTGA |
| **36** | *CgbHLH33.2* | F: CTGGTGGTAGTGCGACTGTT | **97** | *CgbHLH107.2* | F: GCCATTTCATTGGTCCGAGC |
|  |  | R: CCCAGTATTGAAGCCCTAAGCA |  |  | R: TTCCTCATTTCCGCCACCTC |
| **37** | *CgbHLH35* | F: TGATGGAAAACCTCGGCGAA | **98** | *CgbHLH107.3* | F: TGTGAGCTCTGTTCACCAGG |
|  |  | R: CGAAAACGCCTCGTCCAATG |  |  | R: TTGTGCATGTAATCCCAGAGA |
| **43** | *CgbHLH39* | F: TCCCGTTGCAGATCAAACGA | **101** | *CgbHLH112* | F: AGGATCCAGAAGCGCCAAAA |
|  |  | R: ATCTCTCGACTTGCTGCTGG |  |  | R: CAACGGAGACACGTCCTGAA |
| **51** | *CgbHLH48* | F: CCAGAGAACGCCAACTCCTT | **105** | *CgbHLH122* | F: AAGCCACCTACTGGTGCAAA |
|  |  | R: AGTTTGCCCCTGGGTTGAAA |  |  | R: GCCCGTTGAATAATTATGGCCT |
| **52** | *CgbHLH49* | F: AGGAGCCATCTCAGGTAGCC | **106** | *CgbHLH123* | F: TACCGCAGCCACAACAAGAT |
|  |  | R: ATCCGGTGGTGTAGACCCAT |  |  | R: CAGAATCAACAGCAGGGGGT |
| **57** | *CgbHLH62* | F: CGTCAGCCTTTTTCAGCCAC | **107** | *CgbHLH128* | F: AAACGTGGCTGTGCTACTCA |
|  |  | R: GCAGGTCATCCTCACCGAAA |  |  | R: CTGCACTGCCAGGTCTAACA |
| **58** | *CgbHLH63* | F: GGCCAAGCTACTGATAGCCA | **108** | *CgbHLH130.1* | F: AAGACTCTCAGCGACAACCG |
|  |  | R: GCAGCTAGTTTCATCGACAGG |  |  | R: AGCCCTGTACCTTTCTTGGC |
| **59** | *CgbHLH68* | F: ACTGCCTAGCGACGATGATG | **109** | *CgbHLH130.2* | F: GGGAACCAAAGCATGGGGAA |
|  |  | R: ACAAGTTGTTGGCTTGGGGA |  |  | R: TCGCTTGGAAAATTGGGCAT |
| **60** | *CgbHLH69.1* | F: CCTAGCTGCAACAAGACGGA | **114** | *CgbHLH153* | F: CTTGTGCTGTTGACCAAGGC |
|  |  | R: CAGCTGCACCCAGTCTACTC |  |  | R: CTTTCCTCTCCTTGGCGGAG |

**Table S2 The primers of *CgbHLH* genes used for qRT-PCR**
